# Cloning and Characterization of a Flavonol Synthase Gene From *Litchi chinensis* and Its Variation Among Litchi Cultivars With Different Fruit Maturation Periods

**DOI:** 10.3389/fpls.2018.00567

**Published:** 2018-04-25

**Authors:** Wei Liu, Zhidan Xiao, Chao Fan, Nonghui Jiang, Xiangchun Meng, Xu Xiang

**Affiliations:** ^1^Institute of Fruit Tree Research, Guangdong Academy of Agricultural Sciences, Guangzhou, China; ^2^Key Laboratory of South Subtropical Fruit Biology and Genetic Resource Utilization, Ministry of Agriculture, Guangzhou, China; ^3^Guangdong Provincial Key Laboratory of Tropical and Subtropical Fruit Tree Research, Guangzhou, China; ^4^Guangdong Provincial Key Laboratory of Biotechnology for Plant Development, School of Life Sciences, South China Normal University, Guangzhou, China

**Keywords:** *Litchi chinensis* Sonn., flavonol synthase (FLS), allelic diversity, fruit maturation period, extremely early-maturing trait, selection of cultivars

## Abstract

Litchi (*Litchi chinensis*) is an important subtropical fruit tree with high commercial value. However, the short and centralized fruit maturation period of litchi cultivars represents a bottleneck for litchi production. Therefore, the development of novel cultivars with extremely early fruit maturation period is critical. Previously, we showed that the genotypes of extremely early-maturing (EEM), early-maturing (EM), and middle-to-late-maturing (MLM) cultivars at a specific locus SNP51 (substitution type C/T) were consistent with their respective genetic background at the whole-genome level; a homozygous C/C genotype at SNP51 systematically differentiated EEM cultivars from others. The litchi gene on which SNP51 was located was annotated as flavonol synthase (*FLS*), which catalyzes the formation of flavonols. Here, we further elucidate the variation of the *FLS* gene from *L. chinensis* (*LcFLS*) among EEM, EM, and MLM cultivars. EEM cultivars with a homozygous C/C genotype at SNP51 all contained the same 2,199-bp sequence of the *LcFLS* gene. For MLM cultivars with a homozygous T/T genotype at SNP51, the sequence lengths of the *LcFLS* gene were 2,202–2,222 bp. EM cultivars with heterozygous C/T genotypes at SNP51 contained two different alleles of the *LcFLS* gene: a 2,199-bp sequence identical to that in EEM cultivars and a 2,205-bp sequence identical to that in MLM cultivar ‘Heiye.’ Moreover, the coding regions of *LcFLS* genes of other MLM cultivars were almost identical to that of ‘Heiye.’ Therefore, the *LcFLS* gene coding region may be used as a source of diagnostic SNP markers to discriminate or identify genotypes with the EEM trait. The expression pattern of the *LcFLS* gene and accumulation pattern of flavonol from EEM, EM, and MLM cultivars were analyzed and compared using quantitative real-time PCR (qRT-PCR) and high-performance liquid chromatography (HPLC) for mature leaves, flower buds, and fruits, 15, 30, 45, and 60 days after anthesis. Flavonol content and *LcFLS* gene expression levels were positively correlated in all three cultivars: both decreased from the EEM to MLM cultivars, with moderate levels in the EM cultivars. *LcFLS* gene function could be further analyzed to elucidate its correlation with phenotype variation among litchi cultivars with different fruit maturation periods.

## Introduction

Litchi (*Litchi chinensis* Sonn.), a member of the family Sapindaceae, is an important subtropical fruit tree with high commercial value because of the excellent taste and rich nutritional value of its fruits (Li et al., 2013). China has a long history of litchi cultivation (more than 2,300 years), and abundant germplasm resources have been established (Wu, 1998). However, most litchi cultivars ripen between mid-June and mid-July. This results in a short and centralized fruit maturation period, which represents a bottleneck for litchi production in China (Li, 2008). Therefore, development of novel litchi cultivars with extremely early fruit maturation periods is critical for the industry.

The fruit maturation period of litchi is determined jointly by the flowering time and the fruit development process. The molecular mechanisms underlying the fruit maturation period of litchi are not yet fully understood; to our knowledge, there have been only two studies addressing this subject (Zhao et al., 2011; Ding et al., 2015). Zhao et al. (2011) detected five quantitative trait loci for the fruit maturation period using an F_1_ hybrid population from a cross of litchi cultivars: ‘Maguili’ × ‘Jiaohesanyuehong.’ Ding et al. (2015) showed that *LcFT1* played an essential part in litchi floral induction, and that sequence differences in the *LcFT1* promoter may be one of the causes of the natural variation in the flowering times of different litchi cultivars.

Previously, we surveyed the genetic relationships between 96 litchi accessions collected across China, using 90 single nucleotide polymorphisms (SNPs) evenly spaced across the litchi genome (Liu et al., 2015). The 96 litchi accessions could be classified into four main groups according to the fruit maturation period: extremely early-maturing (EEM), early-maturing (EM), middle-maturing, and late-maturing (Liu et al., 2015). Two clusters were detected among the 96 litchi accessions using STRUCTURE analysis. Accessions with the EEM trait were assigned to cluster 1, and accessions with the middle-to-late-maturing (MLM) trait were assigned to cluster 2. Accessions with the EM trait showed an almost equal percentage of admixture with each cluster (Liu et al., 2015). We further compared the genotypes of each SNP locus among these three groups. We identified a specific locus SNP51 (substitution type C/T), at which all six EEM cultivars were the only cultivars that showed a homozygous C/C genotype; all 75 except five MLM cultivars showed a homozygous T/T genotype, whereas all 15 except three EM cultivars showed a heterozygous C/T genotype. Therefore, the genotypes of litchi cultivars with different fruit maturation periods at the locus SNP51 seemed to be consistent with their respective genetic backgrounds on the whole-genome level, as identified by the previous STRUCTURE analysis (Liu et al., 2015). Litchi cultivars with different fruit maturation periods exhibited systematic variation in genotype at SNP51, and the homozygous C/C genotype at SNP51 could be used to systematically differentiate EEM cultivars from the other cultivars.

The gene on which SNP51 is located in litchi was annotated as flavonol synthase (FLS)^1^, which belongs to the 2-oxoglutarate-dependent dioxygenase subfamily. The first *FLS* gene was isolated from *Petunia hybrida* (Holton et al., 1993). More *FLS* genes were cloned and characterized from various plant species, including *Arabidopsis thaliana* (Pelletier et al., 1997; Owens et al., 2008; Preuss et al., 2009), *Eustoma grandiflorum* (Nielsen et al., 2002), *Ginkgo biloba* (Xu et al., 2012), and *Vaccinium corymbosum* (Zhang et al., 2016). FLS is an important enzyme in the flavonoid pathway that catalyzes the formation of flavonols. The flavonol pathway is an important downstream branch of the flavonoid pathway, as there is competition between FLS and dihydroflavonol 4-reductase for the common substrates dihydroflavonols, which leads to the biosynthesis of flavonols and anthocyanins (Davies et al., 2003; Luo et al., 2016). Flavonols are the most abundant subgroup of flavonoids and have important functions in plant growth and development, including regulation of auxin transport (Kuhn et al., 2011; Lewis et al., 2011), modulation of leaf and flower color (Takahashi et al., 2007; Tian et al., 2015), and protection against ultraviolet-B irradiation (Bandurska et al., 2012; Majer et al., 2014).

In this study, we aimed to further elucidate the variation of the *FLS* gene from *L. chinensis* (*LcFLS*) among litchi cultivars with different fruit maturation periods. To this end, we cloned and characterized full-length cDNA and genomic DNA sequences of the *LcFLS* gene. We also examined the allelic diversity of the *LcFLS* gene among the EEM, EM, and MLM cultivars. In addition, the *LcFLS* gene expression patterns and flavonol content of the EEM, EM, and MLM cultivars were analyzed and compared using quantitative real-time PCR (qRT-PCR) and high-performance liquid chromatography (HPLC), respectively. To our knowledge, this is the first report of an FLS from the family Sapindaceae.

## Materials and Methods

### Plant Material

Litchi cultivars grown in the National Litchi Germplasm Gene Bank in the Institute of Fruit Tree Research, Guangdong Academy of Agricultural Sciences (Guangzhou, China), were used in this study.

Genomic DNA and cDNA of the *LcFLS* gene were cloned from young leaves of an EEM cultivar ‘Sanyuehong’ and an MLM cultivar ‘Heiye.’ To explore the sequence variation of *LcFLS* gene sequence among cultivars with different fruit maturation periods, genomic DNA of the *LcFLS* gene was also cloned from young leaves of 27 other litchi cultivars (**Table 1**). A 10-year-old litchi tree with uniform vigor was selected for each cultivar, and young leaves were collected during the spring season of 2015.

**Table 1:** Sequence lengths of the *LcFLS* gene and genotypes at SNP51 of 29 litchi cultivars with different fruit maturation periods.

Number	Cultivar	Origin	Fruit maturation period	Genotype at SNP51	Sequence length of *LcFLS* gene (bp)
1	Sanyuehong	Guangdong	Extremely early-maturing	C/C	2199
2	Yuhebao	Guangxi	Extremely early-maturing	C/C	2199
3	Hemaoli	Yunnan	Extremely early-maturing	C/C	2199
4	Yuanliyihao	Yunnan	Extremely early-maturing	C/C	2199
5	Yuanlierhao	Yunnan	Extremely early-maturing	C/C	2199
6	Yuanyangerhao	Yunnan	Extremely early-maturing	C/C	2199
7	Feizixiao	Guangdong	Early-maturing	C/T	2199+2205
8	Shuidong	Guangdong	Early-maturing	C/T	2199+2205
9	Dazao	Guangdong	Early-maturing	C/T	2199+2205
10	Baitangying	Guangdong	Early-maturing	C/T	2199+2205
11	Guangxitangbo	Guangxi	Early-maturing	C/T	2199+2205
12	Siyuehong	Guangxi	Early-maturing	C/T	2199+2205
13	Lanzhu	Fujian	Early-maturing	C/T	2199+2205
14	Dachenzi	Fujian	Early-maturing	C/T	2199+2205
15	Yuanhong	Fujian	Early-maturing	C/T	2199+2205
16	Heiye	Guangdong	Middle-to-late-maturing	T/T	2205
17	Lvhebao	Fujian	Middle-to-late-maturing	T/T	2202
18	Xinqiumili	Fujian	Middle-to-late-maturing	T/T	2202
19	Edanli	Hainan	Middle-to-late-maturing	T/T	2206
20	Nuomici	Guangdong	Middle-to-late-maturing	T/T	2207
21	Huaizhi	Guangdong	Middle-to-late-maturing	T/T	2207
22	Hongdenglong	Guangdong	Middle-to-late-maturing	T/T	2207
23	Jinzhong	Guangxi	Middle-to-late-maturing	T/T	2207
24	Xiafanzhi	Fujian	Middle-to-late-maturing	T/T	2207
25	Lingshanxiangli	Guangxi	Middle-to-late-maturing	T/T	2211
26	Wuheli	Hainan	Middle-to-late-maturing	T/T	2211
27	Maguili	Guangdong	Middle-to-late-maturing	T/T	2219
28	Huangpili	Hainan	Middle-to-late-maturing	T/T	2221
29	Jizuili	Guangxi	Middle-to-late-maturing	T/T	2222

To compare the expression levels of the *LcFLS* gene and flavonol content among cultivars with different fruit maturation periods, the representative EEM cultivar ‘Sanyuehong,’ EM cultivar ‘Shuidong,’ and MLM cultivar ‘Nuomici’ were used. Mature leaves, flower buds, and fruits of the three cultivars were collected. Mature leaves were collected during the summer season of 2015. Flower buds were collected during the floral differentiation stage; the sampling dates were January 26, February 27, and March 18 of 2015 for ‘Sanyuehong,’ ‘Shuidong,’ and ‘Nuomici,’ respectively. Fruit samples were collected at four representative stages of development and maturation, 15, 30, 45, and 60 days after anthesis (DAA), respectively. Pericarp and seed were separated for fruits at 30 DAA. Pericarp, aril, and seed were separated for fruits at 45 and 60 DAA. Fruitlets at 15 DAA were used as a whole because separation was difficult at this stage. Three replications were set for each cultivar. All samples were frozen in liquid nitrogen upon collection and stored at -80°C until further use.

### Cloning of the Full-Length cDNA and Genomic DNA of the *LcFLS* Gene

The *LcFLS* gene sequences were obtained from the litchi genome database (see foot note text 1). Primers were designed to isolate the *LcFLS* gene and explore its expression patterns (**Table 2**). Genomic DNA was extracted from fresh leaves of litchi cultivars using a modified CTAB method (Murray and Thompson, 1980). Total RNA was extracted from the fresh leaves using an RNAout 2.0 kit (Tiandz, China), and cDNA was synthesized with a PrimeScript^TM^ II 1st Strand cDNA synthesis kit (Takara, Japan). The genomic DNA and cDNA from leaves were used as templates to amplify the *LcFLS* gene from *L. chinensis*. PCR-amplified products were cloned into a pMD19-T vector (Takara, Japan), transformed into *Escherichia coli* DH5α, and then sequenced at the Beijing Genomics Institute (Beijing, China).

**Table 2:** Primers for cloning and for quantitative real-time PCR (qRT-PCR) assay of *LcFLS* gene.

Primer name	Sequence (5′–3′)	Function
LcFLS-F	TAAAAACACTAGAGTGGCCTTGGC	Gene cloning
LcFLS-R	ATAGAATGTCTTTATTTTGGCACGG	Gene cloning
Q LcFLS-F	GCTACACAGAACCACCGTCA	qRT-PCR
Q LcFLS-R	TGGGAGGATTTTCAGGGTCA	qRT-PCR
Q LcActin-F	AGTTTGGTTGATGTGGGAGAC	qRT-PCR
Q LcActin-R	TGGCTGAACCCGAGATGAT	qRT-PCR

### Bioinformatic Analysis

*LcFLS* gene sequences were analyzed using online bioinformatics tools^2^^,^^3^. Multiple sequence alignments were performed with DNAMAN 8.0^4^. The three-dimensional (3D) structure of LcFLS was built using the Web-based SWISS-MODEL server homology modeling pipeline. A phylogenetic tree was constructed using MEGA 7.0 with the neighbor-joining method (Kumar et al., 2016). The reliability of the constructed trees was tested with a bootstrapping method using 1,000 replicates.

### qRT-PCR Analysis

Total RNA from mature leaves, flower buds, and fruits of litchi cultivars were extracted using the RNAout 2.0 kit (Tiandz, China). Total RNA was reverse transcribed into cDNA with the aid of the PrimeScript^TM^ RT reagent kit (Takara, Japan). Transcript levels of the *LcFLS* gene were analyzed using qRT-PCR with a CFX Connect^TM^ Real-Time PCR Detection System (Bio-Rad, United States) and SYBR^®^ Premix Ex Taq^TM^ II (Takara, Japan) according to the manufacturer’s instructions. Specific qRT-PCR primers were designed with Primer Express Software 3.0 (Applied Biosystems, United States) (**Table 2**). Gene expression values were calculated using the 2^-ΔΔCt^ method (Livak and Schmittgen, 2001) and normalized using the *LcActin* gene (GenBank accession number HQ615689) as a reference. All reactions were performed in triplicate (with three biological replicates).

### Determination of Flavonol Content

Harvested organs (mature leaves, flower buds, and fruits) were lyophilized for 48 h and ground into a fine powder. Powdered samples (100 mg) were extracted by adding 80% (v/v) methanol. The samples were then hydrolyzed by adding an equal volume of 2 N HCl, followed by incubation at 70°C for 40 min. During incubation, samples were vortexed every 10 min. After extraction, one volume of 100% (v/v) methanol was added, and the extracts were centrifuged at 12,000 rpm for 20 min. The supernatant was filtered through a 0.22-μm syringe filter and subjected to HPLC analysis. HPLC was performed with a C18 column (250 mm × 4.6 mm, 5 μm; Sunfire, United States) using a Waters Alliance 2695 separation module. Methanol and double-distilled water were used as mobile phases A and B, respectively. The linear elution gradient method was used as follows: 0–5 min, 80% B; 5–30 min, 80% B; 30–40 min, 0% B. The flow rate was maintained at 1.0 mL/min. An injection volume of 10 μL and a wavelength of 368 nm were used for analysis. Quercetin was selected as the standard sample because it was the predominant flavonol in litchi tissue (Guo et al., 2008). Standard quercetin was purchased from Sigma-Aldrich (United States). The compounds in the sample were determined using a standard curve. All analyses were performed with three biological replicates.

## Results

### Identification of the Full-Length cDNA and Genomic DNA Sequences of the *LcFLS* Gene

The full-length cDNA sequence of the *LcFLS* gene amplified from the representative EEM cultivar ‘Sanyuehong’ was 1,079 bp long and contained a 1,008-bp open reading frame (ORF). The full-length genomic DNA of the *LcFLS* gene from ‘Sanyuehong’ was 2,199 bp long and had 100% identity with the coding region of the full-length cDNA sequence. Comparison of the cDNA sequence and the genomic DNA sequence indicated that there were three exons in the *LcFLS* gene, exon 1 (468 bp), exon 2 (327 bp), and exon 3 (213 bp), separated by two introns, intron 1 (459 bp) and intron 2 (661 bp).

**FIGURE 1:**
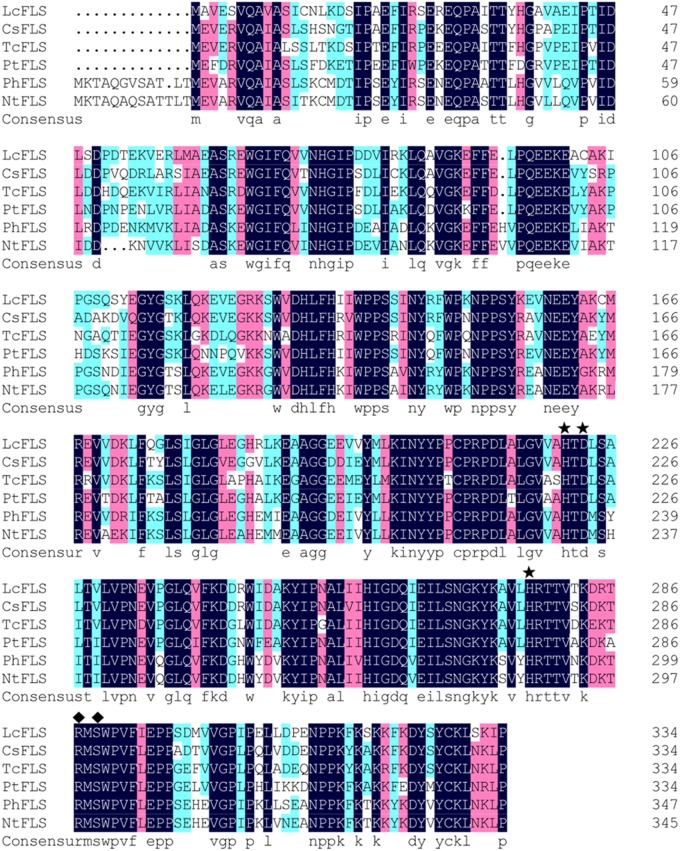
Sequence alignments of *Litchi chinensis* flavonol synthase (LcFLS) and flavonol synthase (FLS) proteins from *Citrus sinensis* (CsFLS), *Theobroma cacao* (TcFLS), *Populus trichocarpa* (PtFLS), *Petunia hybrida* (PhFLS), and *Nicotiana tabacum* (NtFLS); asterisks and diamonds indicate HXD and RXS motifs, respectively.

**FIGURE 2:**
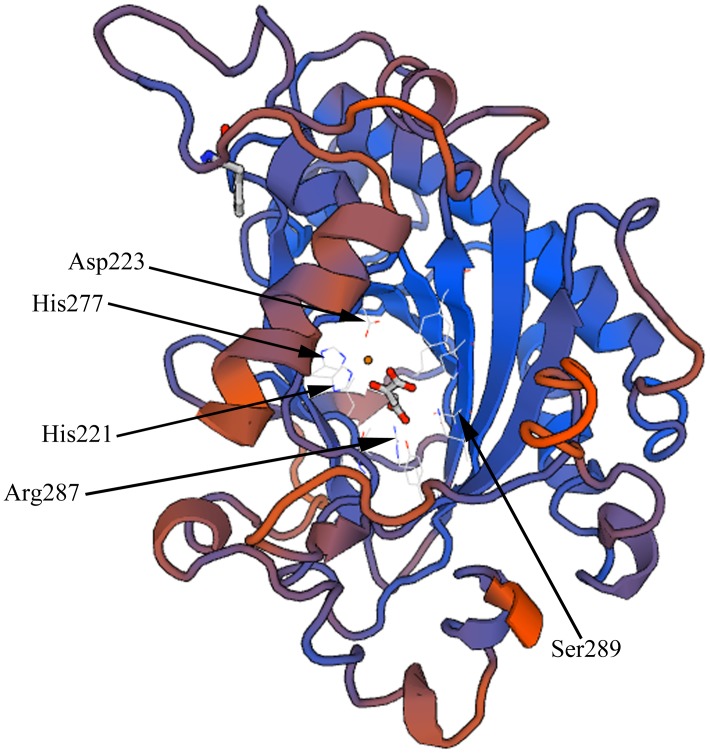
Three-dimensional structures of the deduced LcFLS protein; the conserved ferrous iron-binding sites at His221, Asp223, and His277, and 2-oxoglutarate-binding sites at Arg287 and Ser289 are indicated.

**FIGURE 3:**
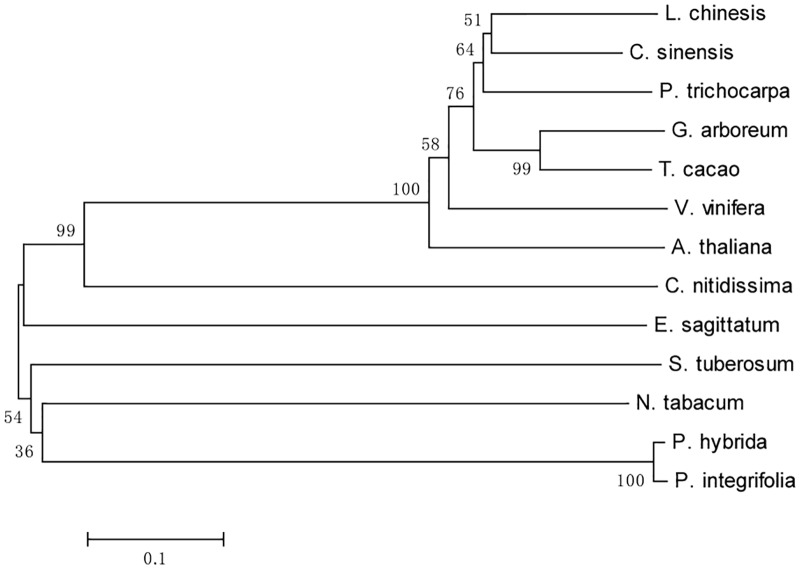
Phylogenetic analysis of LcFLS and FLS proteins from selected species. Numbers at each interior branch indicate bootstrap support of 1,000 replicates. GenBank accession numbers are as follows: *Solanum tuberosum* (NP_001274926), *Camellia nitidissima* (ADZ28516), *Vitis vinifera* (BAE75808), *Epimedium sagittatum* (ABY63659), *Gossypium arboreum* (KHG18898), *Citrus sinensis* (XP_006466183), *Theobroma cacao* (EOY09743), *Populus trichocarpa* (XP_002325697), *Petunia hybrid* (Q07512), *Nicotiana tabacum* (NP_001312508), *Arabidopsis thaliana* (NP_196481), and *Petunia integrifolia* (ALQ80982).

The deduced LcFLS protein contained 335 amino acids, with a calculated molecular weight of 37.88 kDa and an isoelectric point of 5.72. LcFLS had amino acid homology with the previously reported FLS proteins from *Citrus sinensis* (CsFLS, XP_006466183, 78%), *Theobroma cacao* (TcFLS, EOY09743, 74%), *Populus trichocarpa* (PtFLS, XP_002325697, 74%), *P. hybrida* (PhFLS, Q07512, 72%), and *Nicotiana tabacum* (NtFLS, NP_001312508, 70%) (**Figure 1**). A GenBank conserved domain database search revealed that LcFLS belongs to the 2OG-Fe(II) oxygenase superfamily, which is characterized by the presence of a conserved 2OG-FeII Oxy domain. The conserved sequence motifs of other members of the 2OG-FeII oxygenase superfamily, the H × D × nH motif for ligating ferrous iron (His221, Asp223, and His277) and the R × S motif participating in the 2-oxoglutarate binding (Arg287 and Ser289), were also found (**Figure 1**). The 3D structure of LcFLS was generated based on the crystal structure of the *A. thaliana* anthocyanidin synthase (AtANS; PDB ID 1gp5) (Wilmouth et al., 2002). The sequence similarity between LcFLS and the AtANS homologue was 45.18%, and the overall structures of LcFLS and AtANS were very similar (**Figure 2**). Notably, the conserved ferrous iron- and 2-oxoglutarate-binding sites that constitute the active site of LcFLS were found (**Figure 2**). Phylogenetic analysis of the *LcFLS* gene and 10 other *FLS* genes showed that the *FLS* gene of *L. chinensis* is closely related to that of *C. sinensis* (**Figure 3**).

### Allelic Variation in the *LcFLS* Gene Among Litchi Cultivars With Different Fruit Maturation Periods

We amplified the cDNA and genomic DNA of the *LcFLS* gene from the representative MLM cultivar ‘Heiye.’ The full-length cDNA sequence of the *LcFLS* gene amplified from ‘Heiye’ was 1,079 bp long and contained a 1,008-bp ORF. The full-length genomic DNA of the *LcFLS* gene from ‘Heiye’ was 2,205 bp long and had 100% identity with the coding region of its full-length cDNA sequence.

A comparison of the 2,205 bp and 2,199 bp sequences of the *LcFLS* genes from ‘Heiye’ and ‘Sanyuehong’ showed only 95% identity. Researchers from the China Litchi and Longan Industry Technology Research System have completed the whole-genome sequencing of litchi cultivar ‘Feizixiao’ (an EM cultivar), assembled its highly heterozygous diploid genome into two haplotypes^5^, and identified pairs of allelic genes between the two haplotypes (unpublished data). The 2,199-bp and 2,205-bp sequences of the *LcFLS* genes from ‘Sanyuehong’ and ‘Heiye’ identified in our study were found to be two alleles on different haplotypes of a single gene (unpublished data).

To explore the allelic variation of *LcFLS* gene among litchi cultivars with different fruit maturation periods, full-length genomic DNA of the *LcFLS* genes from 27 other litchi cultivars was sequenced; the results are presented in **Table 1**. There were three main findings. (1) For the EEM cultivars with a homozygous C/C genotype at SNP51, the sequence length of the *LcFLS* gene was 2,199 bp, and all the sequences were identical to that of ‘Sanyuehong.’ (2) For the MLM cultivars with a homozygous T/T genotype at SNP51, the sequence length of the *LcFLS* gene ranged from 2,202 to 2,222 bp, and sequences with the same length were identical. The length variation was attributed mainly to the number variation of simple sequence repeat motif (AT)n in the first intron in *LcFLS* genes (**Supplementary Figure S1**). (3) All EM cultivars with a heterozygous C/T genotype at SNP51 contained two different alleles of the *LcFLS* gene: a 2,199-bp sequence identical to those in all EEM cultivars, and a 2,205-bp sequence identical to that in MLM cultivar ‘Heiye.’

**FIGURE 4:**
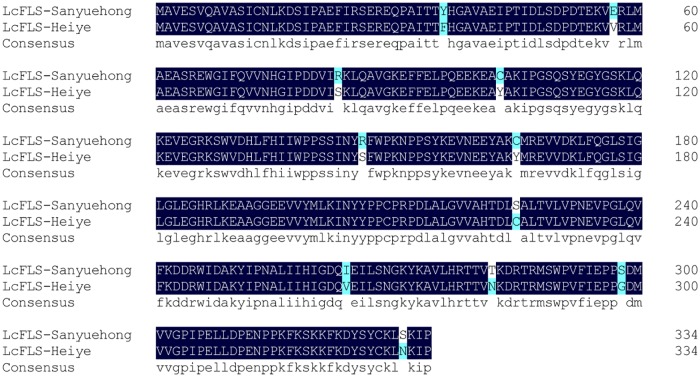
Sequence alignment between open reading frame (ORF) of extremely early-maturing (EEM) cultivar ‘Sanyuehong’ and middle-to-late-maturing (MLM) cultivar ‘Heiye.’

A comparison of the full-length genomic DNA sequence of the *LcFLS* gene from other MLM cultivars with the ORF of the *LcFLS* gene from ‘Heiye’ showed that the sequences were almost identical, except for one non-synonymous SNP in each of exon 1 and exon 3, and two synonymous SNPs in exon 2 (**Table 3**). Therefore, the ORFs of the *LcFLS* gene in litchi cultivars could be divided into two types: the ORF of EEM cultivar ‘Sanyuehong’ and the ORF of MLM cultivar ‘Heiye.’ A comparison of the ORFs of the *LcFLS* genes of Sanyuehong’ and ‘Heiye’ gave 27 SNPs, including 16 synonymous and 11 non-synonymous SNPs (**Figure 4**). The 3D structure of LcFLS from ‘Heiye’ was similar to that of ‘Sanyuehong’ (result not shown).

**Table 3:** Nucleotide diversity of the *LcFLS* gene open reading frame (ORF) for the middle-to-late-maturing (MLM) cultivar ‘Heiye’ (*LcFLS*-2205 bp) and other MLM cultivars (*LcFLS*-2,202 bp to 2,222 bp).

*LcFLS* gene	Exon 1	Exon 2	Exon 3
*LcFLS*-2202bp	–	ACG-ACC (Thr-Thr)	–
*LcFLS*-2206bp	–	ACG-ACC (Thr-Thr)	GTG-ATG (Val-Met)
*LcFLS*-2207bp	–	ACG-ACC (Thr-Thr)	–
*LcFLS*-2211bp	TTT-TAT (Phe-Tyr)	ACG-ACC (Thr-Thr)	–
*LcFLS*-2219bp	TTT-TAT (Phe-Tyr)	ACG-ACC (Thr-Thr), GTG-GTA (Val-Val)	–
*LcFLS*-2221bp	TTT-TAT (Phe-Tyr)	ACG-ACC (Thr-Thr)	–
*LcFLS*-2222bp	TTT-TAT (Phe-Tyr)	ACG-ACC (Thr-Thr)	–

### *LcFLS* Gene Expression Profiles in Litchi Cultivars With Different Fruit Maturation Periods

**FIGURE 5:**
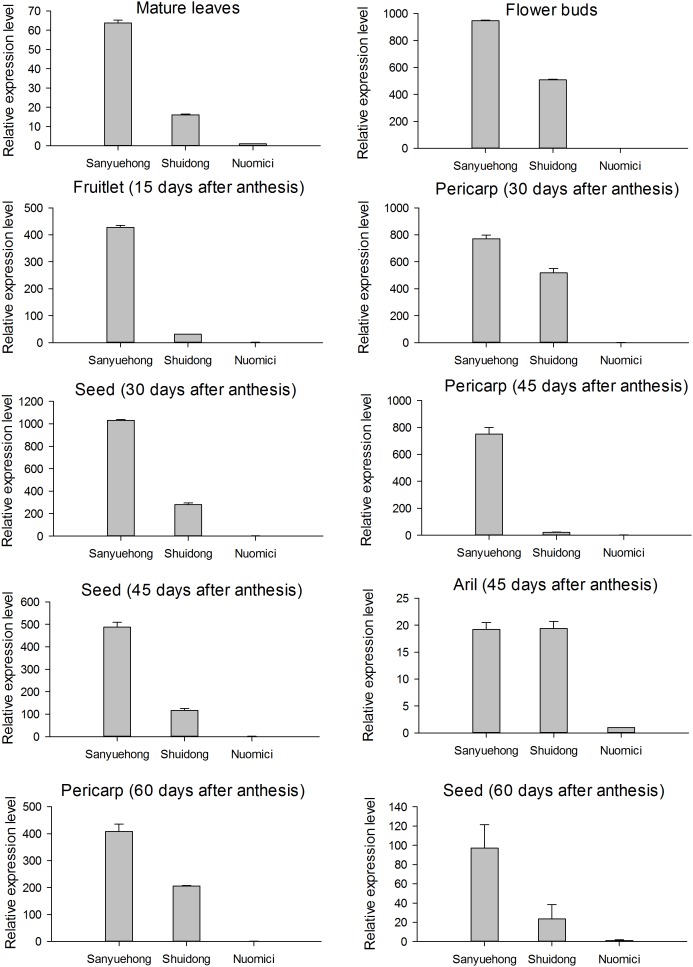
Expression profile of the *LcFLS* gene among various tissues of EEM cultivar ‘Sanyuehong,’ early-maturing (EM) cultivar ‘Shuidong’ and MLM cultivar ‘Nuomici’; vertical bars represent the standard error of triplicate experiments.

To compare the expression patterns of the *LcFLS* gene among litchi cultivars with different fruit maturation periods, the transcript levels of *LcFLS* genes in mature leaves, flower buds, and fruits of the EEM cultivar ‘Sanyuehong,’ EM cultivar ‘Shuidong,’ and MLM cultivar ‘Nuomici’ were analyzed using qRT-PCR (**Supplementary Data Sheet S1**). The transcript levels of the *LcFLS* gene were generally highest in ‘Sanyuehong,’ whereas they were lowest in ‘Nuomici’ (**Figure 5**).

In the mature leaves, *LcFLS* transcript levels were 63.8- and 16.1-fold higher in ‘Sanyuehong’ and ‘Shuidong,’ respectively, than in ‘Nuomici.’ The *LcFLS* gene transcript level in flower buds reached a 946.1- and 508.7-fold higher level in ‘Sanyuehong’ and ‘Shuidong,’ respectively, than in ‘Nuomici.’ In the fruitlets at 15 DAA, *LcFLS* transcript levels were 427.6- and 31.2-fold higher in ‘Sanyuehong’ and ‘Shuidong,’ respectively, than in ‘Nuomici.’ Pericarp and seed were separated for the fruits of 30 DAA; the expression levels of the *LcFLS* gene in these two tissues were 771.0- and 1031.1-fold higher in ‘Sanyuehong’ than in ‘Nuomici.’ In the fruits of 45 DAA, pericarp, aril, and seed were separated; the *LcFLS* gene showed the highest expression levels in pericarp and seed of ‘Sanyuehong’ and the lowest level in those of ‘Nuomici.’ In the case of aril, *LcFLS* mRNA levels were similar in ‘Sanyuehong’ and ‘Shuidong,’ and approximately 19-fold higher than in ‘Nuomici.’ For the pericarp of fruits at 60 DAA, *LcFLS* transcript levels were 407.9- and 205.6-fold higher in ‘Sanyuehong’ and ‘Shuidong,’ respectively, than that in ‘Nuomici.’ No *LcFLS* expression could be detected in the aril of fruits at 60 DAA.

### Flavonol Content in Litchi Cultivars With Different Fruit Maturation Periods

To further examine the correlation between flavonol content and *LcFLS* expression levels among litchi cultivars with different fruit maturation periods, the content of quercetin (a major type of flavonol in litchi tissue) was also measured for the EEM cultivar ‘Sanyuehong,’ EM cultivar ‘Shuidong,’ and MLM cultivar ‘Nuomici’ using HPLC analysis.

**FIGURE 6:**
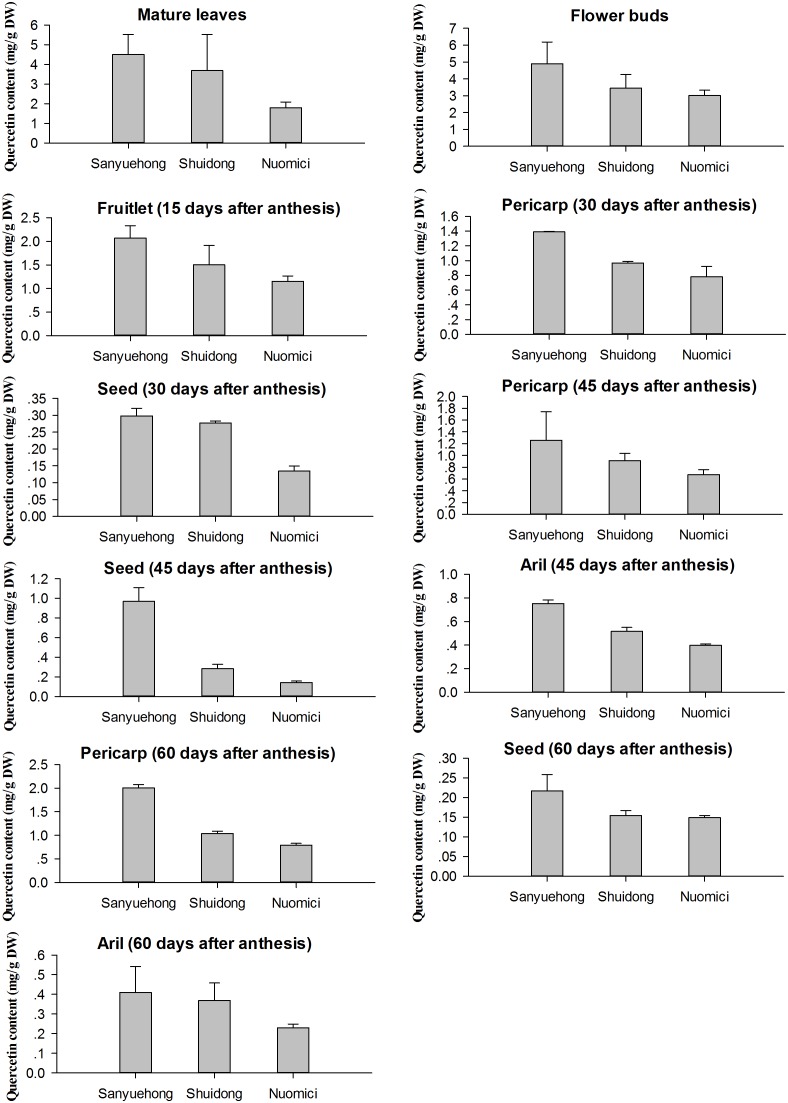
Quercetin content in various tissues of EEM cultivar ‘Sanyuehong,’ early-maturing (EM) cultivar ‘Shuidong,’ and MLM cultivar ‘Nuomici’; values are the means of three independent experiments, calculated as mg quercetin equivalent per 1 g dry weight (mg/g).

The correlation coefficient (*R*^2^) between different concentrations of quercetin standards and their peak areas was greater than 0.99, which indicated that the HPLC method developed in our study has good linearity. The profiles of quercetin standard samples are listed in **Supplementary Figure S2**. The quercetin content of various tissues of three litchi cultivars is shown in **Figure 6**. The quercetin content was highest in the EEM cultivar ‘Sanyuehong,’ with moderate levels in the EM cultivar ‘Shuidong’ and the lowest amounts in the MLM cultivar ‘Nuomici.’ The accumulation patterns of quercetin in the three litchi cultivars correlated well with the transcription patterns of the *LcFLS* gene.

## Discussion

### Allelic Variation in the *LcFLS* Gene Among Litchi Cultivars With Different Fruit Maturation Periods

Owing to the long juvenile period of litchi, conventional natural breeding and cross-breeding are time-consuming. Predicting the fruit maturation period of individuals at an early stage, using molecular markers closely associated with the desirable trait, should reduce the space and labor investment and greatly accelerate the progress of litchi breeding trials. In our previous study, we found a perfect correlation between litchi phylogenetic relationships and the fruit maturation period, based on 90 SNPs evenly spaced across the litchi genome (Liu et al., 2015). We identified a specific locus SNP51 (substitution type C/T), genotypes at which of EEM cultivars (all C/C), EM cultivars (mostly C/T), and MLM cultivars (mostly T/T) seemed to be completely in accordance with their genetic background on the whole-genome level; moreover, the homozygous C/C genotype at SNP51 systematically differentiated EEM cultivars from others. In the present study, we further elucidated variation in the gene on which SNP51 is located – that is, FLS of litchi (*LcFLS*) – among the EEM, EM, and MLM cultivars. To our knowledge, this is the first report of an FLS from the family Sapindaceae.

The full-length genomic DNA sequences of the *LcFLS* gene from the EEM cultivar ‘Sanyuehong’ and MLM cultivar ‘Heiye’ were 2,199 bp and 2,205 bp long, respectively, and the two sequences were confirmed to correspond to two alleles on different haplotypes of a single gene (unpublished data). When we compared the allelic variation of *LcFLS* gene among 29 litchi cultivars with different fruit maturation periods (**Table 1**), we found that EEM cultivars with a homozygous C/C genotype at SNP51 all contained a 2,199-bp sequence of the *LcFLS* gene, the same as in ‘Sanyuehong.’ For the MLM cultivars with a homozygous T/T genotype at SNP51, the *LcFLS* gene length ranged from 2,202 to 2,222 bp; this length variation could be attributed mainly to the number variation of simple sequence repeat motif (AT)n in the first intron. Researchers from the China Litchi and Longan Industry Technology Research System have analyzed the genome sizes of 14 litchi accessions using flow cytometry and found significant variation in genome size, ranging from 550 to 620 Mb (unpublished data). Within-species variation in genome size among different accessions has been reported in various species (Biémont, 2008); this variation could be attributed to the different numbers of various repeated sequences, including satellite DNA (Ma and Jackson, 2006; Bosco et al., 2007). Our results suggested that addition and loss of satellite repeat elements might have contributed to the significant differences in genome size found among litchi accessions. All of the EM cultivars with a heterozygous C/T genotype at SNP51 contained two different alleles of *LcFLS* gene, a 2,199-bp sequence identical to that in all EEM cultivars and a 2,205-bp sequence identical to that in MLM cultivar ‘Heiye’. Furthermore, although the genomic DNA sequences of MLM cultivars varied greatly, their coding regions were almost identical to that of ‘Heiye.’ Therefore, there were two different types of ORF sequences of *LcFLS* genes among litchi cultivars, and these two ORF sequences contained 11 non-synonymous SNPs. Several active site residues have previously been identified, including Gly68 and Gly261, which are involved in proper folding of the FLS polypeptide (Wellmann et al., 2002), and H132, F134, K202, F293, and E295, which have roles in substrate binding of the FLS enzyme (Chua et al., 2008). Further studies are required to determine whether the 11 non-synonymous SNPs between the two different LcFLS ORFs lead to different catalytic activities of the FLS enzyme between litchi cultivars with different fruit maturation periods.

Based on comparison of the allelic variation of the *LcFLS* gene among EEM, EM, and MLM cultivars, we proposed that the coding region of the *LcFLS* gene represents a source of diagnostic SNP markers (such as SNP51) to discriminate or identify genotypes with the EEM trait in litchi. Liu (2001) analyzed the genetic relationships of 60 Chinese litchi accessions using 470 random amplified polymorphic DNA (RAPD) loci and found that these accessions could be classified into three groups, which corresponded to the traits of EEM, EM, and late-to-extremely late-maturing. Liu (2001) identified 18 RAPD loci that were present only in EEM and EM cultivars, but absent in all late-to-extremely late-maturing cultivars. Huang (2005) subsequently converted one of the 18 RAPD loci to a sequence-characterized amplified region (SCAR) marker named SC-Em-J06-1360 and suggested that this marker could be used for early selection for breeding of litchi cultivars with EEM and EM traits. However, the 18 RAPD loci identified by Liu (2001) and the SCAR marker developed by Huang (2005) could not discriminate EEM cultivars from EM cultivars. To the best of our knowledge, our study is the first to identify a litchi gene and SNP marker that may be used for reliable and robust discrimination of EEM cultivars from others.

### *LcFLS* Gene Expression Profile and Accumulation Pattern of Flavonol Among Litchi Cultivars With Different Fruit Maturation Periods

The expression pattern of the *LcFLS* gene and the accumulation pattern of flavonol (quercetin) from EEM, EM, and MLM cultivars were analyzed and compared using qRT-PCR and HPLC. We found positive correlations between flavonol (quercetin) content and *LcFLS* gene expression level among these three cultivars, indicating that the *LcFLS* gene encodes one of the key enzymes in the flavonol biosynthesis pathway of *L. chinensis*, which might be responsible for the formation of quercetin in *L. chinensis*. Positive correlations between flavonoid concentration and mRNA levels of *FLS* genes have also been reported in other plant species. For example, Moriguchi et al. (2002) isolated an *FLS* gene from *Satsuma mandarin* (*Citrus unshiu* Marc.), *CitFLS*, and found that increases in its transcript levels in fruit peel correlated with flavonol accumulation during fruit development. Fujita et al. (2006) obtained genomic sequences of *FLS* genes from *Vitis vinifera* ‘Cabernet Sauvignon’ (*VvFLSs*) and found that the accumulation pattern of quercetin coincided with the change in mRNA accumulation of the *VvFLSs* gene in the berry skins. Moreover, Xu et al. (2012) investigated tissue-specific and development-dependent transcript levels of the *FLS* gene of *G. biloba* (*GbFLS*) and found that the expression pattern of *GbFLS* correlated with the accumulation pattern of flavonols. Li et al. (2012) cloned and characterized an *FLS* gene from *Fagopyrum tataricum* (*FtFLS*), revealing an organ-specific expression pattern of the *FtFLS* gene that corresponded with trends in flavonoid content.

Among the three litchi cultivars with different fruit maturation periods, the transcript levels of the *LcFLS* gene and flavonol (quercetin) content both decreased from the EEM cultivar ‘Sanyuehong’ to the MLM cultivar ‘Nuomici,’ with moderate levels in the EM cultivar ‘Shuidong.’ In plants, flavonols are significantly involved in plant growth and development, and have been identified as the most active flavonoids, acting as regulators of polar auxin transport (Jacobs and Rubery, 1988; Buer et al., 2013; Yin et al., 2014). Reduction of quercetin content in *FLS*-silenced tobacco lines has been shown to increase polar auxin transport toward the root, decreasing the levels of auxin at apical region of shoots and thus leading to delayed flowering (Mahajan et al., 2011). In addition, flavonols usually act as co-pigments, affecting the color of many plant organs (Aida et al., 2000; Nielsen et al., 2002), and can also impart color by themselves (Middleton and Teramura, 1993). For example, overexpression of *Camellia nitidissima FLS1* (*CnFLS1*) in tobacco altered the floral color to white or light yellow, and metabolic analysis indicated a significant increase in flavonol levels in transgenic plants, suggesting that *CnFLS1* has a critical role in yellow color pigmentation (Zhou et al., 2013).

In litchi, the major distinguishing characteristic among the EEM, EM, and MLM cultivars is their fruit maturation period. EEM cultivars generally have earlier flowering dates and a shorter fruit development process (Li, 2008). Flavonols are known to be key regulators of auxin transport (Jacobs and Rubery, 1988; Buer et al., 2013; Yin et al., 2014), and auxin has been shown to have an important role in determining flowering time (Mahajan et al., 2011) and in the fruit development process (Mounet et al., 2012; Dare et al., 2013; El-Sharkawy et al., 2014). Thus, the flavonols might be involved in the differences in fruit maturation periods observed among litchi cultivars through the regulation of auxin transport. The color of flower buds is another distinguishing characteristic among the cultivars. EEM, EM, and MLM cultivar flower buds are dark brown, brown, and light-yellow, respectively (Liu, 2001); however, the mechanisms underlying this difference have never been explored. The decreasing flavonol content from EEM to EM and MLM cultivars might contribute to the gradually lightening color of their flower buds. Furthermore, there might be other characteristics among EEM, EM, and MLM litchi cultivars that have not yet been discovered. Therefore, future research should focus on functional analysis of the *LcFLS* gene and elucidation of its correlation with distinctive characteristics among litchi cultivars with different fruit maturation periods.

## Author Contributions

WL and XX conceived and designed the study. ZX conducted the experiments. CF, NJ, and XM prepared the plant material and performed the analysis. WL, ZX, and XX wrote and revised the manuscript. All authors read, reviewed, and approved the manuscript.

## Conflict of Interest Statement

The authors declare that the research was conducted in the absence of any commercial or financial relationships that could be construed as a potential conflict of interest.

## Footnotes

1 http://litchidb.genomics.cn/page/species/index.jsp

2 http://www.expasy.org

3 http://www.ncbi.nlm.nih.gov

4 http://www.lynnon.com

5 http://litchidb.genomics.cn

## Supplementary material

FIGURE S1 Sequence alignments of the first intron of *LcFLS* genes cloned from middle-to-late-maturing (MLM) cultivars.

FIGURE S2 The chromatogram of quercetin standard samples.**(A)** 0.005 mg/mL; **(B)** 0.01 mg/mL; **(C)** 0.02 mg/mL; **(D)** 0.04 mg/mL; and **(E)** 0.08 mg/mL.

DATA SHEET S1 Raw quantitative real-time PCR (qRT-PCR) data.

## References

[B1] Aida R. Yoshida K. Kondo T. Kishimoto S. Shibata M. (2000). Copigmentation gives bluer flowers on transgenic torenia plants with the antisense dihydroflavonol-4-reductase gene. *Plant Sci.* 160 49–56. 10.1016/S0168-9452(00)00364-2 11164576

[B2] Bandurska H. Pietrowska-Borek M. Cieślak M. (2012). Response of barley seedlings to water deficit and enhanced UV-B irradiation acting alone and in combination. *Acta Physiol. Plant.* 34 161–171. 10.1007/s11738-011-0814-9

[B3] Biémont C. (2008). Genome size evolution: within-species variation in genome size. *Heredity* 101 297–298. 10.1038/hdy.2008.80 18665185

[B4] Bosco G. Campbell P. Leiva-Neto J. T. Markow T. A. (2007). Analysis of Drosophila species genome size and satellite DNA content reveals significant differences among strains as well as between species. *Genetics* 177 1277–1290. 10.1534/genetics.107.075069 18039867 PMC2147996

[B5] Buer C. S. Kordbacheh F. Truong T. T. Hocart C. H. Djordjevic M. A. (2013). Alteration of flavonoid accumulation patterns in transparent testa mutants disturbs auxin transport, gravity responses, and imparts long-term effects on root and shoot architecture. *Planta* 238 171–189. 10.1007/s00425-013-1883-3 23624937

[B6] Chua C. S. Biermann D. Goo K. S. Sim T.-S. (2008). Elucidation of active site residues of *Arabidopsis thaliana* flavonol synthase provides a molecular platform for engineering flavonols. *Phytochemistry* 69 66–75. 10.1016/j.phytochem.2007.07.006 17719613

[B7] Dare A. P. Tomes S. Jones M. McGhie T. K. Stevenson D. E. Johnson R. A. (2013). Phenotypic changes associated with RNA interference silencing of chalcone synthase in apple (*Malus × domestica*). *Plant J.* 74 398–410. 10.1111/tpj.12140 23398045

[B8] Davies K. M. Schwinn K. E. Deroles S. C. Manson D. G. Lewis D. H. Bloor S. J. (2003). Enhancing anthocyanin production by altering competition for substrate between flavonol synthase and dihydroflavonol 4-reductase. *Euphytica* 131 259–268. 10.1023/A:1024018729349

[B9] Ding F. Zhang S. W. Chen H. B. Su Z. X. Zhang R. Xiao Q. S. (2015). Promoter difference of LcFT1 is a leading cause of natural variation of flowering timing in different litchi cultivars (*Litchi chinensis* Sonn.). *Plant Sci.* 241 128–137. 10.1016/j.plantsci.2015.10.004 26706065

[B10] El-Sharkawy I. Sherif S. M. Jones B. Mila I. Kumar P. P. Bouzayen M. (2014). TIR1-like auxin-receptors are involved in the regulation of plum fruit development. *J. Exp. Bot.* 65 5205–5215. 10.1093/jxb/eru279 24996652 PMC4157706

[B11] Fujita A. Goto-Yamamoto N. Aramaki I. Hashizume K. (2006). Organ-specific transcription of putative flavonol synthase genes of grapevine and effects of plant hormones and shading on flavonol biosynthesis in grape berry skins. *Biosci. Biotechnol. Biochem.* 70 632–638. 10.1271/bbb.70.632 16556978

[B12] Guo C. J. Xu J. Wei J. Y. Yang J. J. Wu J. Q. (2008). The flavonoid content of common fruits in China. *Acta Nutr. Sin.* 30 130–135.

[B13] Holton T. A. Brugliera F. Tanaka Y. (1993). Cloning and expression of flavonol synthase from *Petunia hybrida*. *Plant J.* 4 1003–1010. 10.1046/j.1365-313X.1993.04061003.x 7904213

[B14] Huang T. L. (2005). *Molecular Marker Analysis of Two Important Traits of Litchi chinensis Sonn and Genetic Linkage Map Construction of Dimocarpus longan Lour.* Ph.D. dissertation, South China Agricultural University, Guangzhou.

[B15] Jacobs M. Rubery P. H. (1988). Naturally occurring auxin transport regulators. *Science* 241 346–349. 10.1126/science.241.4863.346 17734864

[B16] Kuhn B. M. Geisler M. Bigler L. Ringli C. (2011). Flavonols accumulate asymmetrically and affect auxin aransport in Arabidopsis. *Plant Physiol.* 156 585–595. 10.1104/pp.111.175976 21502189 PMC3177260

[B17] Kumar S. Stecher G. Tamura K. (2016). MEGA7: molecular evolutionary genetics analysis version 7.0 for bigger datasets. *Mol. Biol. Evol.* 33 1870–1874. 10.1093/molbev/msw054 27004904 PMC8210823

[B18] Lewis D. R. Ramirez M. V. Miller N. D. Vallabhaneni P. Ray W. K. Helm R. F. (2011). Auxin and ethylene induce flavonol accumulation through distinct transcriptional networks. *Plant Physiol.* 156 144–164. 10.1104/pp.111.172502 21427279 PMC3091047

[B19] Li C. L. Bai Y. C. Li S. J. Chen H. Han X. Y. Zhao H. X. (2012). Cloning, characterization, and activity analysis of a flavonol synthase gene FtFLS1 and its association with flavonoid content in tartary buckwheat. *J. Agric. Food Chem.* 60 5161–5168. 10.1021/jf205192q 22563787

[B20] Li C. Q. Wang Y. Huang X. M. Li J. Wang H. C. Li J. G. (2013). De novo assembly and characterization of fruit transcriptome in *Litchi chinensis* Sonn and analysis of differentially regulated genes in fruit in response to shading. *BMC Genomics* 14:552. 10.1186/1471-2164-14-552 23941440 PMC3751308

[B21] Li J. G. (2008). *The litchi.* Beijing: China Agriculture Press.

[B22] Liu C. M. (2001). *Construction of Genetic Linkage Map and Analysis of Germplasm Resources of Litchi chinensis Soon.* Ph.D. dissertation, South China Agricultural University, Guangzhou.

[B23] Liu W. Xiao Z. D. Bao X. L. Yang X. Y. Fang J. Xiang X. (2015). Identifying litchi (*Litchi chinensis* Sonn.) cultivars and their genetic relationships using single nucleotide polymorphism (SNP) markers. *PLoS One* 10:e0135390. 10.1371/journal.pone.0135390 26261993 PMC4532366

[B24] Livak K. J. Schmittgen T. D. (2001). Analysis of relative gene expression data using real-time quantitative PCR and the 2-ΔΔC_t_ method. *Methods* 25 402–408. 10.1006/meth.2001.1262 11846609

[B25] Luo P. Ning G. Wang Z. Shen Y. Jin H. Li P. (2016). Disequilibrium of flavonol synthase and dihydroflavonol-4-reductase expression associated tightly to white vs. red color flower formation in plants. *Front. Plant Sci.* 6:1257. 10.3389/fpls.2015.01257 26793227 PMC4710699

[B26] Ma J. Jackson S. A. (2006). Retrotransposon accumulation and satellite amplification mediated by segmental duplication facilitate centromere expansion in rice. *Genome Res.* 16 251–259. 10.1101/gr.4583106 16354755 PMC1361721

[B27] Mahajan M. Ahuja P. S. Yadav S. K. (2011). Post-transcriptional silencing of flavonol synthase mRNA in tobacco leads to fruits with arrested seed set. *PLoS One* 6:e28315. 10.1371/journal.pone.0028315 22145036 PMC3228754

[B28] Majer P. Neugart S. Krumbein A. Schreiner M. Hideg É. (2014). Singlet oxygen scavenging by leaf flavonoids contributes to sunlight acclimation in *Tilia platyphyllos*. *Environ. Exp. Bot.* 100 1–9. 10.1016/j.envexpbot.2013.12.001

[B29] Middleton E. M. Teramura A. H. (1993). The role of flavonol glycosides and carotenoids in protecting soybean from ultraviolet-B damage. *Plant Physiol.* 103 741–752. 10.1104/pp.103.3.741 12231976 PMC159044

[B30] Moriguchi T. Kita M. Ogawa K. Tomono Y. Endo T. Omura M. (2002). Flavonol synthase gene expression during citrus fruit development. *Physiol. Plant.* 114 251–258. 10.1034/j.1399-3054.2002.1140211.x 11903972

[B31] Mounet F. Moing A. Kowalczyk M. Rohrmann J. Petit J. Garcia V. (2012). Down-regulation of a single auxin efflux transport protein in tomato induces precocious fruit development. *J. Exp. Bot.* 63 4901–4917. 10.1093/jxb/ers167 22844095 PMC3427993

[B32] Murray M. G. Thompson W. F. (1980). Rapid isolation of high molecular weight plant DNA. *Nucleic Acids Res.* 8 4321–4326. 10.1093/nar/8.19.4321 7433111 PMC324241

[B33] Nielsen K. Deroles S. C. Markham K. R. Bradley M. J. Podivinsky E. Manson D. (2002). Antisense flavonol synthase alters copigmentation and flower color in *lisianthus*. *Mol. Breed.* 9 217–229. 10.1023/a:1020320809654

[B34] Owens D. K. Alerding A. B. Crosby K. C. Bandara A. B. Westwood J. H. Winkel B. S. J. (2008). Functional analysis of a predicted flavonol synthase gene family in Arabidopsis. *Plant Physiol.* 147 1046–1061. 10.1104/pp.108.117457 18467451 PMC2442520

[B35] Pelletier M. K. Murrell J. R. Shirley B. W. (1997). Characterization of flavonol synthase and leucoanthocyanidin dioxygenase genes in Arabidopsis. Further evidence for differential regulation of “early” and “late” genes. *Plant Physiol.* 113 1437–1445. 10.1104/pp.113.4.1437 9112784 PMC158268

[B36] Preuss A. Stracke R. Weisshaar B. Hillebrecht A. Matern U. Martens S. (2009). *Arabidopsis thaliana* expresses a second functional flavonol synthase. *FEBS Lett.* 583 1981–1986. 10.1016/j.febslet.2009.05.006 19433090

[B37] Takahashi R. Githiri S. M. Hatayama K. Dubouzet E. G. Shimada N. Aoki T. (2007). A single-base deletion in soybean flavonol synthase gene is associated with magenta flower color. *Plant Mol. Biol.* 63 125–135. 10.1007/s11103-006-9077-z 17006592

[B38] Tian J. Han Z. Zhang J. Hu Y. Song T. Yao Y. (2015). The balance of expression of dihydroflavonol 4-reductase and flavonol synthase regulates flavonoid biosynthesis and red foliage coloration in crabapples. *Sci. Rep.* 5:12228. 10.1038/srep12228 26192267 PMC4507444

[B39] Wellmann F. Lukacin R. Moriguchi T. Britsch L. Schiltz E. Matern U. (2002). Functional expression and mutational analysis of flavonol synthase from *Citrus unshiu*. *Eur. J. Biochem.* 269 4134–4142. 10.1046/j.1432-1033.2002.03108.x 12180990

[B40] Wilmouth R. C. Turnbull J. J. Welford R. W. D. Clifton I. J. Prescott A. G. (2002). Structure and mechanism of anthocyanidin synthase from *Arabidopsis thaliana*. *Structure* 10 93–103. 10.1016/S0969-2126(01)00695-5 11796114

[B41] Wu S. X. (1998). *Encyclopedia of China fruits.* Beijing: China Forestry Press.

[B42] Xu F. Li L. L. Zhang W. W. Cheng H. Sun N. N. Cheng S. Y. (2012). Isolation, characterization, and function analysis of a flavonol synthase gene from Ginkgo biloba. *Mol. Biol. Rep.* 39 2285–2296. 10.1007/s11033-011-0978-9 21643949

[B43] Yin R. Han K. Heller W. Albert A. Dobrev P. I. Zažímalová E. (2014). Kaempferol 3-O-rhamnoside-7-O-rhamnoside is an endogenous flavonol inhibitor of polar auxin transport in Arabidopsis shoots. *New Phytol.* 201 466–475. 10.1111/nph.12558 24251900 PMC4260840

[B44] Zhang C. Y. Liu H. C. Jia C. G. Liu Y. J. Wang F. T. Wang J. Y. (2016). Cloning, characterization and functional analysis of a flavonol synthase from *Vaccinium corymbosum*. *Trees* 30 1595–1605. 10.1007/s00468-016-1393-6

[B45] Zhao Y. H. Guo Y. S. Fu J. X. Huang S. S. Lu B. B. Zhou J. (2011). Molecular genetic map construction and QTL analysis for fruit maturation period in litchi. *Biotechnol. Biotechnol. Equip.* 25 2315–2320. 10.5504/bbeq.2011.0046

[B46] Zhou X. W. Fan Z. Q. Chen Y. Zhu Y. L. Li J. Y. Yin H. F. (2013). Functional analyses of a flavonol synthase-like gene from *Camellia nitidissima* reveal its roles in flavonoid metabolism during floral pigmentation. *J. Biosci.* 38 593–604. 10.1007/s12038-013-9339-2 23938391

