# Supplementary material for: Cloning and Characterization of a Flavonol Synthase Gene From Litchi chinensis and Its Variation Among Litchi Cultivars With Different Fruit Maturation Periods
Source: Front Plant Sci. 2018 Apr 25;9:567. doi: 10.3389/fpls.2018.00567 (PMC5996885; doi:10.3389/fpls.2018.00567)
Supplement: FIGURE S1 — Sequence alignments of the first intron of LcFLS genes cloned from middle-to-late-maturing (MLM) cultivars. [file Presentation_1.PDF]

Supplementary Material

Cloning and Characterization of a Flavonol Synthase Gene from *Litchi chinensis* and Its Variation among Litchi Cultivars with Different Fruit Maturation Periods

Wei Liu[1,2,3#], Zhidan Xiao[4#], Chao Fan[1,2,3], Nonghui Jiang[1,2,3], Xiangchun Meng[1,2,3], Xu Xiang[1,2,3*]

*** Correspondence:** Corresponding Author: xiangxu@vip.163.com

1 Supplementary Figures

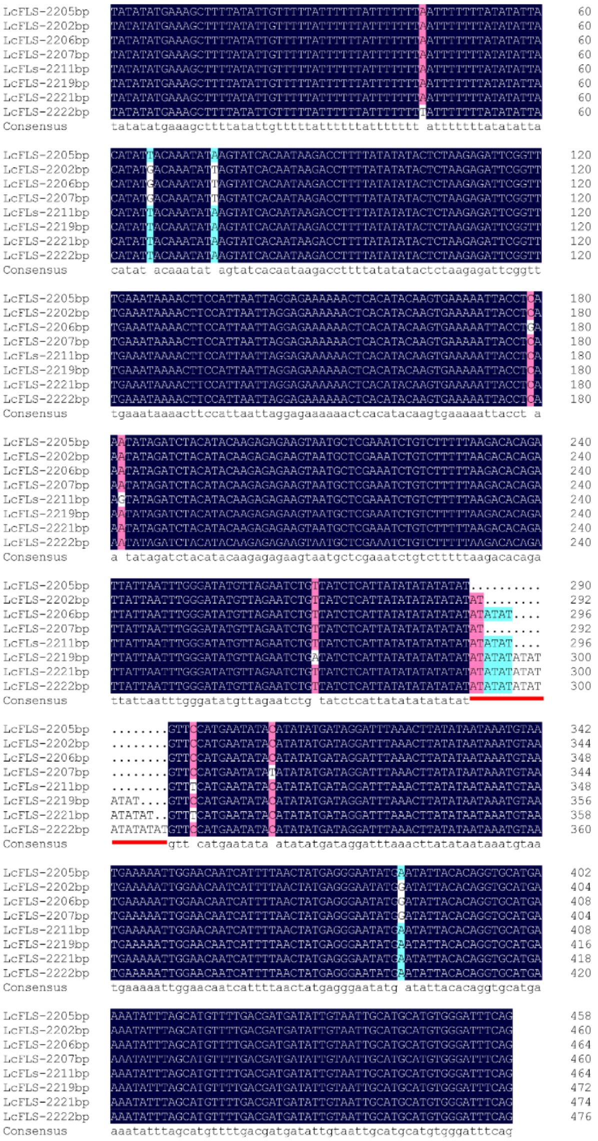

Supplementary Figure 1. Sequence alignments of the first intron of *LcFLS* genes cloned from middle-to-late-maturing (MLM) cultivars.

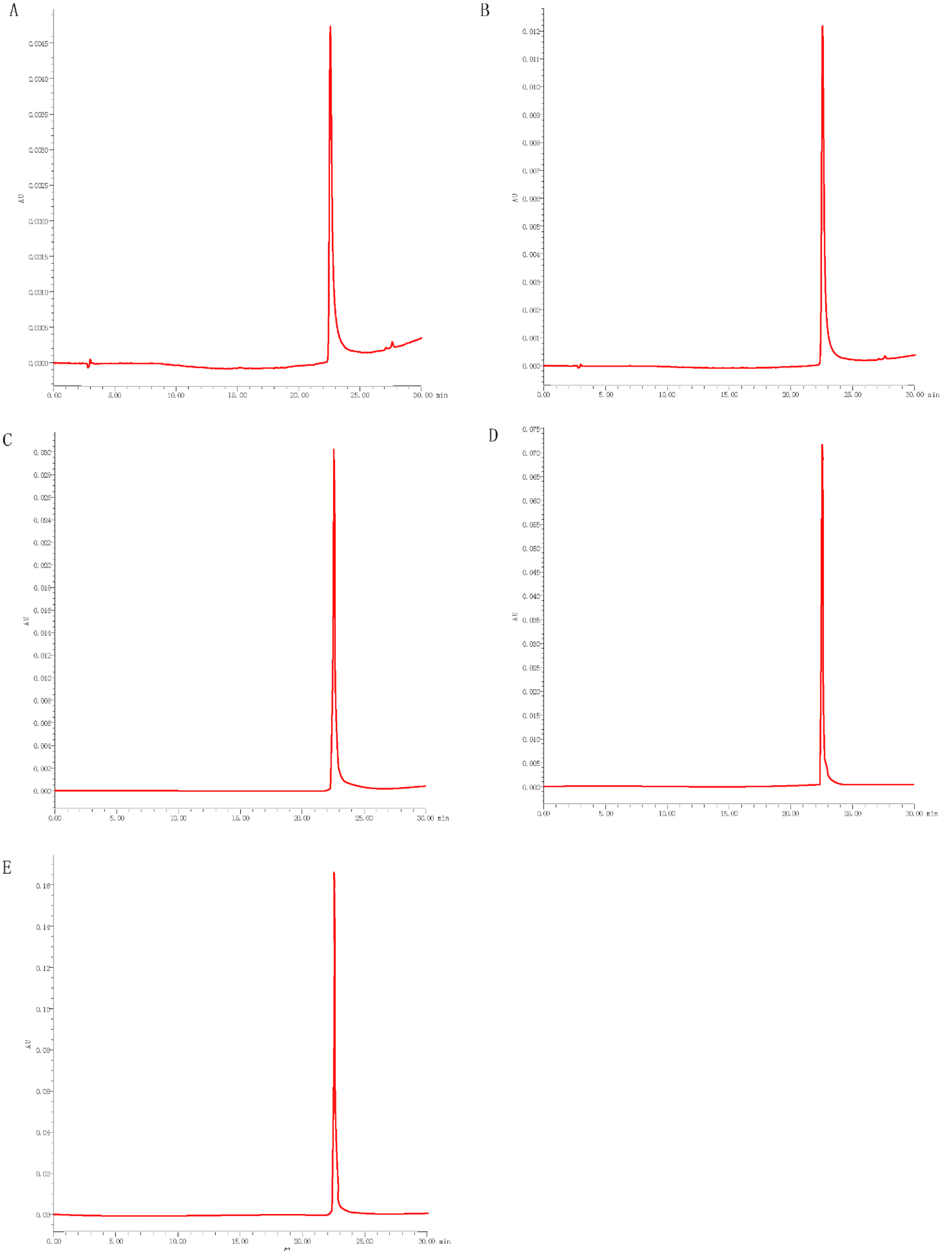
A
B
C
D
E
AU
min

Supplementary Figure 2. The chromatogram of quercetin standard samples. (A) 0.005 mg/mL; (B) 0.01 mg/mL; (C) 0.02 mg/mL; (D) 0.04 mg/mL; (E) 0.08 mg/mL.
